# Supplementary material for: Novel αO-conotoxin GeXIVA[1,2] Nonaddictive Analgesic with Pharmacokinetic Modelling-Based Mechanistic Assessment
Source: Pharmaceutics. 2022 Aug 26;14(9):1789. doi: 10.3390/pharmaceutics14091789 (PMC9505004; doi:10.3390/pharmaceutics14091789)
Supplement: Supplementary file 1 [file pharmaceutics-14-01789-s001.zip › pharmaceutics-1850455-supplementary.pdf]

Table S1. Summary of mean PWT value of oxaliplatin-induced neuropathic pain rats after IM administered 0.11, 0.22 and 0.44 mg·kg^{-1} GeXIVA[1,2]

Time (h)	Observed PWT value (g)		
	0.11 mg·kg-1	0.22 mg·kg-1	0.44 mg·kg-1
0	2.58	2.79	2.73
1	4.66	4.86	6.19
2	7.08	6.40	12.32
4	6.63	11.39	14.53
6	3.84	4.72	8.40

Table S2 Observed (n= 13 or 14) and model simulated PD effect of intramuscular 0.11, 0.22 and 0.44 mg·kg^{-1} GeXIVA[1,2]

Dose	0.44 mg·kg^{-1}			0.22 mg·kg^{-1}			0.11 mg·kg^{-1}		
Parameter	Pred	Obs	Ratio	Pred	Obs	Ratio	Pred	Obs	Ratio
Time(h)									
0	2.28	2.73	1.20	2.28	2.79	1.22	2.28	2.58	1.13
1	5.53	6.19	1.12	5.27	4.86	0.92	4.87	4.66	0.96
2	10.52	12.32	1.17	9.18	6.40	0.70	7.47	7.08	0.95
4	16.71	14.53	0.87	10.76	11.39	1.06	6.88	6.63	0.96
6	9.90	8.40	0.85	6.11	4.72	0.77	4.17	3.84	0.92
AUC (g·h)	65.77	63.49	1.04	47.81	43.37	1.10	35.15	33.68	1.04

(A)

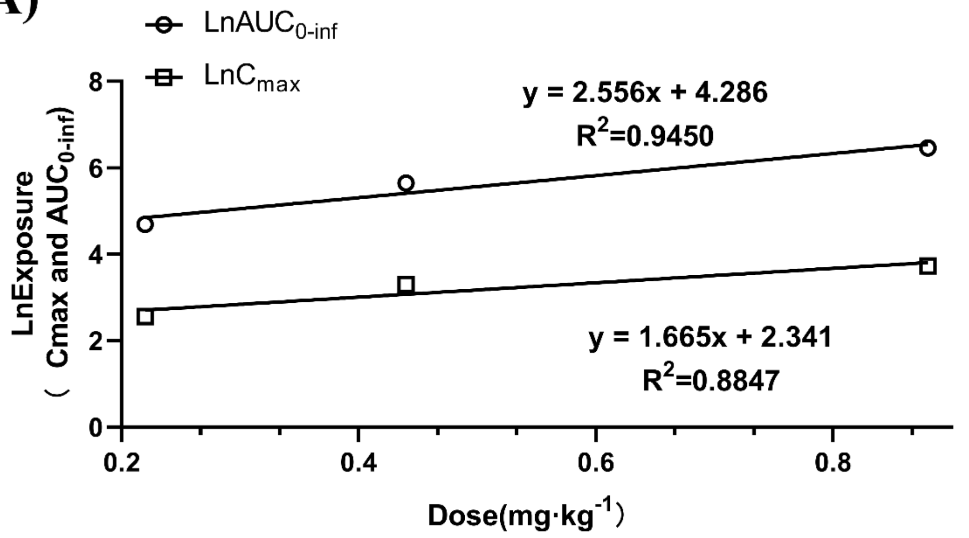

(B)

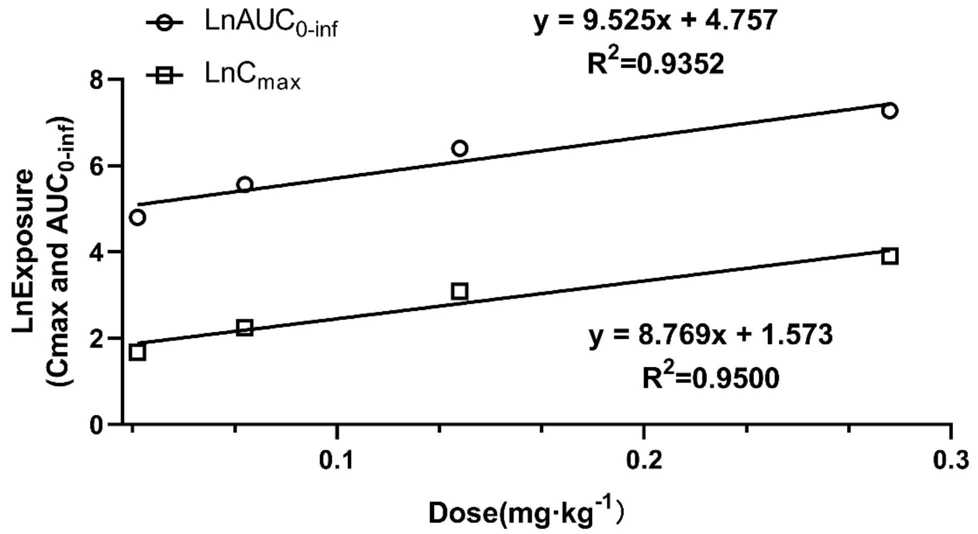

Figure S1 Linear regression of Ln C_{max} and Ln $AUC_{0–inf}$ versus dose for GeXIVA[1,2]. (A) in SD rats after an IM dose of 0.22, 0.44, 0.88 mg·kg^{-1}; (B) in beagle dogs after an IM dose of 0.035, 0.07, 0.14 or 0.28 mg·kg^{-1}
